# An Experimental Method for Stereolithic Mandible Fabrication and Image Preparation

**DOI:** 10.2174/1874120700701010004

**Published:** 2007-07-17

**Authors:** Shawn Russett, Paul Major, Jason Carey, Roger Toogood, Pierre Boulanger

**Affiliations:** a Department of Orthodontics, University of Alberta, Edmonton, Alberta Canada; b Department of Engineering, University of Alberta, 4-9 Mechanical Engineering Building, Edmonton, Alberta T6G 2G8, Canada; c Department of Engineering, University of Alberta, Edmonton, Alberta T6G 2G8, Canada; d Department of Computing Science, University of Alberta, Edmonton, Alberta, Canada

**Keywords:** Biomedical engineering, Rapid prototyping, Image processing, Stereolithic mandible models.

## Abstract

Reproduction of anatomical structures by rapid prototyping has proven to be a valid adjunct for craniofacial surgery, providing alternative methods to produce prostheses and development of surgical guides. The aim of this study was to introduce a methodology to fabricate asymmetric human mandibles by rapid prototyping to be used in future studies for evaluating mandibular symmetries. Stereolithic models of human mandibles were produced with varying amounts of asymmetry in the condylar neck, ramus and body of the mandible by means of rapid prototyping. A method for production of the synthetic mandibles was defined. Model preparation, landmark description and development of the experimental model were described. A series of synthetic mandibles ranging in asymmetry were accurately produced from a scanned human mandible. A method for creating the asymmetries, fabricating, coating and landmarking the synthetic mandibles was formulated. A description for designing a reproducible experimental model for image acquisition was also outlined. Production of synthetic mandibles by stereolithic modeling is a viable method for creating skeletal experimental models with known amounts of asymmetry.

## INTRODUCTION

Fabrication of prototype models is a staple of engineering research and design as an intermediate step for developing concepts or inventions and bringing new ideas to fruition. Often small scale models are produced to provide a relatively inexpensive visual and physical connection to aid in the exploration of new ideas. In addition, prototypes are used to assess the feasibility of the design as well as its intricacies and subtleties while avoiding excessive costs and unexpected fabrication flaws in the final product. Rapid prototyping (RP) by means of fused deposition modeling (FDM) is an example of such prototype development. This process of rapid prototyping generates a plastic model from a stereolithic (STL) computer file of the conceptualized object through computer guided plastic extrusion. A heated plastic filament is extruded through the nozzle and deposited onto a platform in layers building a three-dimensional (3-D) plastic model from the bottom up as each layer of plastic cools. The level of intricacy and amount of detail is driven by the information within the original stereolithic computer file as well as the software and hardware settings of the system. When applying this process to model human and animal tissue as a means to replicate biological structures, it can simply be referred to as biomodeling. Biomodeling is a relatively new concept that is quickly gaining momentum for research as a topic of interest over the past decade.

In this short period of time, there have been few areas of research within the medical field that have been explored. Some of the more interesting uses for biomodeling within medical sciences include the reproduction of anatomical structures and biologic anomalies for the purpose of educating patients and guiding surgery [1,2]. In D’Urso’s studies, they were able to reproduce models of tumors and other anomalies to help describe the anatomical areas of interest and the proposed surgical procedures to their patients. In one study, displaying versatility of use, the authors produced RP plastic models of fetal faces that were derived from 3-D ultrasound images [3]. Others were able to produce a replica model of an ear for the purpose of designing a prosthesis [4]. Much of the research has focused on craniofacial surgery and reconstructive surgical planning procedures [5,6,7,8]. RP modeling has also been demonstrated as a useful tool for design and implementation of distraction appliances for the purpose of distraction osteogenesis procedures [9]. From a dental perspective, production of surgical splints, by means of STL modeling, as a surgical guide for implant placement has also been explored [10].

The accuracy of the models produced has been a topic of exploration more recently as it is of little use to have a method to reproduce anatomical structures if they are not dimensionally accurate. Barker *et al.* developed a study to compare the dimensional accuracy of a rapid prototyping technique using stereolithography (SLA) to a dry human skull. The authors found that there was a dimensional accuracy of 97.7-99.12% [11]. In a study published in 1988, Santler *et al.* found that 80% of the STL models they produced were within ± 1mm [12]. Others conducted a study that compared 16 linear measurements made on a dry human skull to the same 16 linear measurements of a rapid prototype replica of the skull. The results indicated that the absolute mean deviation was 0.62 ± 0.35 mm (0.56 ± 0.39%) [13].

A good number of orthodontic patients have mandibular asymmetries that must be corrected. In the field of orthodontics, it is critical that accurate measurements of mandibular asymmetry be made prior to deciding on patient treatment using conventional imaging techniques such as digital panoramic radiographs. In an attempt to critically evaluate the usefulness for measuring asymmetric mandibular shapes as well as linear and angular measurements from digital panoramic radiographs, a technique for producing STL mandibles with known amounts of asymmetry was the primary purpose for developing this methodology.

## METHODOLOGY

**Fig. (1):**
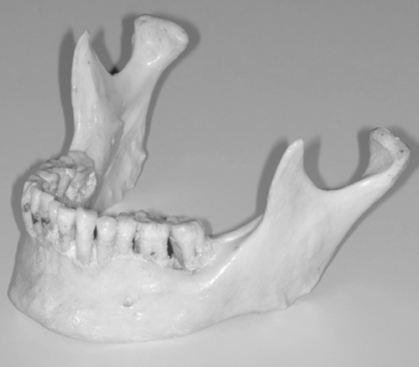
Original skull mandible.

To critically evaluate in further studies the usefulness for measuring asymmetric mandibular shapes as well as linear and angular measurements from digital panoramic radiographs, a technique for producing STL mandibles with known amounts of asymmetry was devised from by scanning an original skull mandible (Fig. **1**) and artificial asymmetries were produced using a CAD software. The following section describes the methodology used.

### Scanning Skull Mandible

The preliminary information required to generate the synthetic mandibles was obtained by scanning the original skull mandible as seen in (Fig. **1**) using a Zephyr^®^ 3-D non-touch laser scanner (Kreon model KZ 50, Limoges, France). The Zephyr^®^ KZ 50 was mounted on a Faro^®^ arm, Titanium series (Kreon, Limoges, France). The Zephyr^®^ laser scanner registers up to 28,800 points per second with a resolution of up to 10 um and a measurement frequency of 60 images per second with 480 points per image. The Faro^®^ arm, Titanium series, is a six axis mounting arm with an accuracy of 12 um. The arm assembly allowed for a convenient and efficient method of capturing the surface images of the skull mandible with a high resolution and accuracy given the combined specifications.

As per the manufacturer’s description, the laser scanner consisted of two components: the laser and a video camera. The laser projected a red line onto the surface of the object of interest to define the surface topography over its length and the video camera recorded the field of view and reflected light intensity as it passed by. The recording is digitized in real time over the entire surface of the object, which results in information that was a 3-D point cloud data set. Through the surface sweeping process and collection of multiple 3-D point sets, a 3-D model is obtained. Fig. (**2**) is a representation of the scanned 3-D model. The laser image was captured on the proprietary software Polygonia^®^ (Kreon, Limoges, France) which is capable of generating multiple files including Initial Graphics Exchange Specification (IGES) and, as in this experiment, stereolithic (STL) files. The original mandible was subsequently stored safely until completion of the project.

### Generating Virtual Mandible and Asymmetries

The STL files generated by the Polygonia^®^ software program were then transferred into Pro/ENGINEER Wildfire 2.0 (PTC, Needham, USA) software program for detailed manipulation and further generation of the mandibular asymmetries. Fig. (**3**) is Pro/ENGINEER triangulated raw data image of the STL file imported from the scanned mandible.

Using the Pro/ENGINEER software, the original STL file was subsequently exported as a solid form using a shrink wrap function in Pro/ENGINEER. This feature converts the virtual mandible from a triangulated surface meshwork into a solid by filling in voids and imperfections by blending the data that was delivered from the Polygonia^®^ software program. The shrink-wrapped model, now a solid file, was imported back into Pro/ENGINEER where normal functions for manipulating solid models could be utilized. The solid model was then sectioned in half. The section was constructed through the dental midline extending through the chin prominence producing a separate left and right mandibular section. Fig. (**4**) represents an image of the virtual mandible divided into left and right halves.

The left half of the model was removed from the file. The corresponding right half of the mandible was used for the remainder of the project. Semi-spherical concavities were designed into the remaining right half of the virtual model as future landmark locations. Fig. (**5**) represents the remaining half (right) of the virtual mandible with concavities for future landmark balls to be inserted. The concavities were designed to accept and securely seat 1.588mm steel balls for landmarks.

**Fig. (6):**
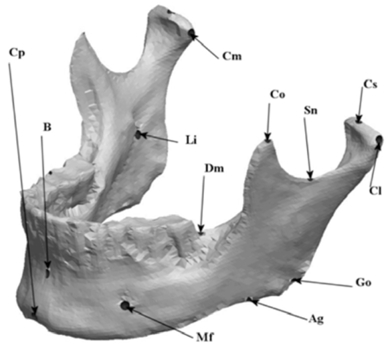
Labeled virtual symmetric mandible.

The remaining virtual half-mandible was then mirror imaged and re-attached to deliver a perfectly symmetric precisely landmarked synthetic mandible and stored as an STL file for fabrication. Labeled virtual mandible and landmark description are shown in (Fig. **6**) and Table **1**, respectively.

The mandible halves were then re-separated and manipulated to create the remaining ranges of asymmetries. The left half of the mandible was sectioned in three areas to which the asymmetries were assigned. Fig. (**7**) shows the locations of the cuts made to the condyle, ramus and the body of the mandible. The Condyle section was located half way between the condylar head and the depth of the sigmoid notch. The location for the cut was determined by constructing a plane half way between the most superior point on the condyle head (Cs) and the depth of the sigmoid notch (Sn). The plane was perpendicular to the Cs-Sn line and was made straight through the neck of the condyle.

**Fig. (10):**
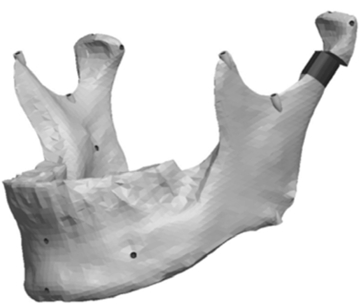
Model with a complex vertical and horizontal lateral condyle asymmetry.

Fig. (**8**) depicts the location in the condylar neck for the sections made to generate the condyle asymmetries. From this section, vertical and complex asymmetries were constructed in the condylar region. The vertical manipulation created was to a maximum of 9mm asymmetry on 3mm increments and the complex condylar asymmetries were 9mm vertical and 6mm horizontal lateral asymmetry with 3mm vertical and 2mm lateral increments. Fig. (**9**) and Figure **10** represent images of models with vertical condylar asymmetry and models with the complex vertical and horizontal lateral condylar asymmetry respectively.

The Body section was located 43mm anterior to Cs as a plane perpendicular to the occlusal plane. The cut extended 8mm into the body before extending 10mm anterior at a 90° angle. The cut then continued vertically at 90° through the remaining body of the mandible forming a “Z-pattern” type cut. Fig. (**11**) outlines the location and pattern of the section made to the body of the mandible to create the body asymmetry. From this section an anteroposterior asymmetry of up to 9mm were constructed in the body region with 3mm increments. Fig. (**12**) represents a model with anteroposterior body asymmetry.

The Ramus section was located half way between the superior aspect of the condylar head and the depth of the antigonial notch. The location for the cut was determined by constructing a point half way between the most superior point on the condyle head (Cs) and the depth of the antigonial notch (Ag). A plane was selected perpendicular to the Cs-Ag line and plane MD which was used to guide the cut that penetrated through the neck of the condyle running parallel to the occlusal plane. Fig. (**13**) represents the location in the ramus of the mandible for the sections made to generate the ramus asymmetries. From this section, vertical and complex asymmetries were constructed in the ramal region. The vertical manipulation studied was to a maximum of 9mm asymmetry on 3mm increments and the complex ramal asymmetry was maximum of 9mm vertical with 3mm increments and 6mm horizontal lateral asymmetries with 2mm increments Fig. (**14**) and Fig. (**15**) represent images of models with the vertical ramal asymmetry and with the complex vertical and horizontal lateral ramal asymmetry respectively.

### Mandible Fabrication

The STL files of the virtual mandibles were programmed into a rapid prototyping (RP) machine (Stratasys^TM^ FDM 8000, Eden Prairie, MA, USA) to generate the STL plastic replica models from the virtual file. This is a process from which the virtual mandible is transformed into a physical mandible occurring by means of fabrication of a plastic model through fused deposition modeling (FDM). In other words, an RP machine is programmed using the pre-established STL program file to feed acrylonitrile-butadiene-styrene (ABS) plastic "wire" through a heated extrusion head where it is melted and deposited in the required pattern. Each pattern delivers the pre-programmed asymmetric mandible desired to an accuracy of 0.62 ± 0.35 mm (0.56 ± 0.39%) [13]. The RP machine was provided by the Department of Mechanical Engineering, University of Alberta in Edmonton, Alberta, Canada.

### Coating and Landmarking Synthetic Mandibles

The constructed, synthetic mandible surface was inspected for gross imperfections, which were removed using a slow speed turbine handpiece (Kavo, Biberach, Germany) and # 2 round latch attachment dental bur (Brassler, USA). Each mandible was then coated with an opaque paint to enable detection by radiographic imaging. The opaque paint consisted of a mixture of 100 ml of Crayola^®^ (Easton, PA, USA) washable non-toxic white paint with 50 mg of Barium Sulfate (BaSO_4_). Due to the fabrication process, the RP models were quite porous and this porosity allowed the custom paint to penetrate beneath the surface. Each mandible was coated with the paint four times to ensure uniform consistency and adequate opacity. The landmarks used in the experiment were 1.588 mm diameter, 316 stainless steel grade100 balls (Small Parts Inc, Miami Lakes, FL, USA). Each landmark position on the synthetic mandibles was identified and the steel balls were fastened into place using cyanoacrylate (Instant Krazy Glue^®^ New York, USA) as per Table 1.

### Experimental Model

**Fig. (16):**
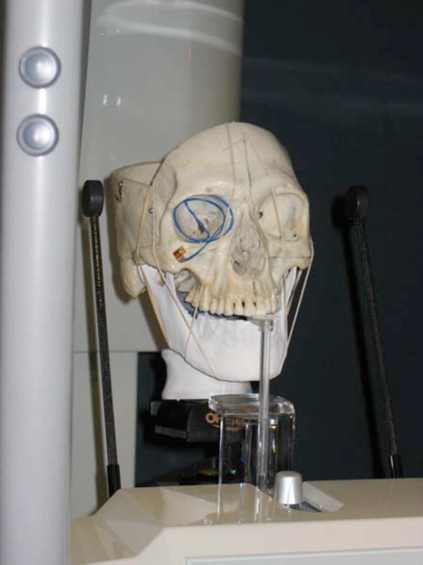
experimental raid prototyped model (mandible) setup in Cephalometric device.

The experimental model (Fig. **16**) was constructed in the following manner using the original skull based with intact maxilla and complete maxillary dentition. The maxillary dentition and fabricated, coated and landmarked mandibular jaws were occluded into a clasp-free morphologically sensitive inter-occlusal thermoset plastic splint. The splint approximated the lower posterior teeth into a protruded position by positioning the anterior teeth in an edge to edge incisor position with an anterior gap of 4mm vertical and 8mm wide for the insertion of the panoramic unit’s bite block. The splint acted to hold the maxilla and each one of the series of synthetic mandibles in a secure and reproducible position throughout the experiment. The splint was constructed using IMPAK^®^ (CMP Industries, Albany, NY, USA) elastic acrylic resin. The temporomandibular joints were positioned onto a uniformly thick 3mm synthetic disc which approximated the joint space. The artificial disc was constructed of Regisil^®^ (Dentsply, York, PA, USA) bite registration material. The disc was fabricated to allow for translation, rotation and lateral movement of position within the glenoid fossa as the asymmetry changed while approximately maintaining the 3mm joint space. The skull and each positioned mandibles were mounted onto an OT-S28V camera tripod (Opus^®^ Ontario, Canada) using a custom designed mounting assembly. The custom mounting assembly consisted of a piece of 76.2 mm long by 38.1 mm diameter, 3 mm gauge polyvinyl tubing that was fastened to a Denar^®^ (Waterpik Technologies, Fort Collins, CO, USA) cast mounting ring. The ring was mounted to the tubing using hot glue resin sticks (3M^TM^ Caulk, Ca, USA). To reproduce the relative position of a patient’s neck and posture, the assembly was attached to the skull over the foramen magnum using the same hot glue resin. The hot glue resin was used to facilitate ease in removal from the skull at project completion. The Denar^®^ mounting ring threaded firmly to the mounting screw supplied with the camera tripod. The assembly allowed for portability and reproducible positioning into a variety of radiographic imaging machines including panoramic and cephalometric units.

## RESULTS

A series of synthetic mandibles ranging in asymmetry were successfully produced from a scanned human mandible. A method for creating the asymmetries, fabricating, coating and landmarking the synthetic mandibles was formulated. Rapid prototyped models with varying degree of asymmetry were produced.

The dimensional accuracy and position of the landmarks was verified using a Cone Beam Computed Tomography (NewTom 3G Scanner, Aperio Services, Verona, Italy) that was previously validated [14]. Experimental marker positions compared to computer model defined positions was excellent. The left side horizontal (Mf-Ag), oblique (Mf-Sn, Mf-Cs) and vertical (Ag-Sn, Ag-Cs and Sn-CS) lengths were identified and measured for true distance three times on separate occasions one week apart by a co-researcher (ML) using the CBCT and the AmiraTM software program. Each set of three measurements on the six lengths were averaged and the average distance obtained was recorded as the true linear distance for each length studied on the synthetic models. The same six linear measurements were collected for both the left and right sides on each of the 35 TIFF digital radiographic images obtained per model. Model accuracy and image magnification factors were established accepting the NewTom® 3G (CBCT) and AmiraTM software as the gold standard of measurement for this project. The CBCT and AmiraTM software were previously reported to measure distances in millimeters with an accuracy of 0.6mm with a measurement error between 0.2 and 0.3mm [14].

**Fig. (17):**
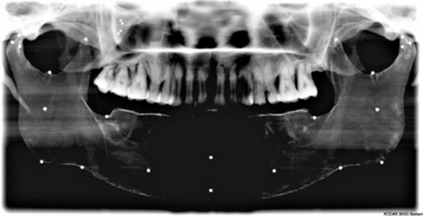
Cephalometric image of experimental model showing rapid prototype geometry and markers.

Experimental models were imaged or scanned using digital panoramic, cephalometric and cone beam computed tomography; geometric features and markers were clearly visible in all images. An example is provided in (Fig. **17**).

## DISCUSSION

The method developed to fabricate an anatomically realistic human mandible by means of rapid prototyping was described. The resultant model proved to be suitable for imaging using digital panoramic, cephalometric and cone beam computed tomography (CBCT). Methodology for providing surface finish, landmarking and development of a reproducible experimental model were also described. The mandibles were constructed as a plastic model by rapid prototyping using STL files programmed into a FDM printing machine. Interestingly, there were various means to obtain STL files suitable for the production of synthetic objects available as resources. Some authors have used 3-D computed tomography image files and converted them to STL files to fabricate models [15,16]. The methodology used in this project employed a non-touch laser scanner. Other authors have used similar technology to scan anatomical parts for production of prosthetic replacements [17]. The ability to use laser scanning techniques enabled efficient collection of surface data and easy conversion to STL files. Manipulation of the STL files that were obtained from the laser scanner were successfully altered using an engineering program to generate the series of asymmetric mandibles. The asymmetric mandibles were produced for future experiments including linear, angular and shape analysis projects.

## CONCLUSION

Fabrication of a series of thirty asymmetric mandibles and one symmetric mandible suitable for imaging by various radiological techniques was established. It was determined that asymmetries in the condyle, ramus and body of the mandible can be successfully designed using engineering software and fabricated by rapid prototyping to construct a STL model of a human mandible. The methodology developed in this paper was planned for use in projects to determine shape changes, linear differences and angular changes between the left and right sides of the synthetic mandibles employing a variety of imaging techniques.

## Figures and Tables

**Fig. (2):**
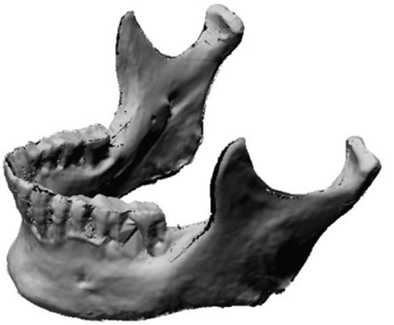
Three-dimensional scan of original mandible.

**Fig. (3):**
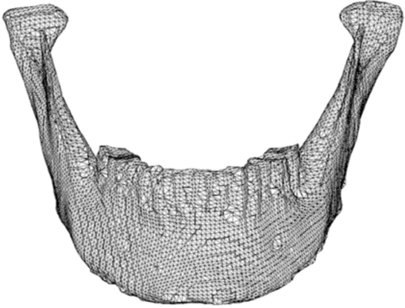
Pro/ENGINEER triangulated STL file.

**Fig. (4):**
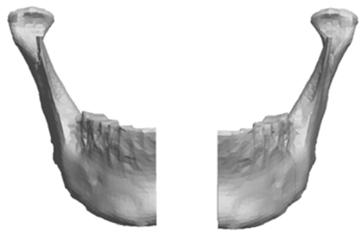
Image of virtual mandible split in half.

**Fig. (5):**
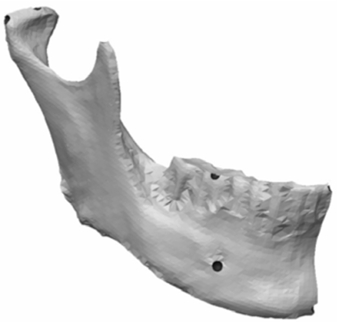
Remaining half of mandible with landmark concavities designed.

**Fig. (7):**
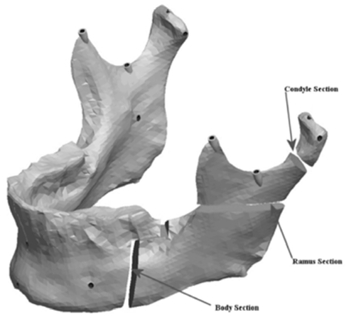
Mandible indicating the cuts for asymmetry.

**Fig. (8):**
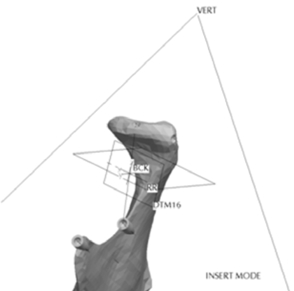
Location of condyle asymmetries.

**Fig. (9):**
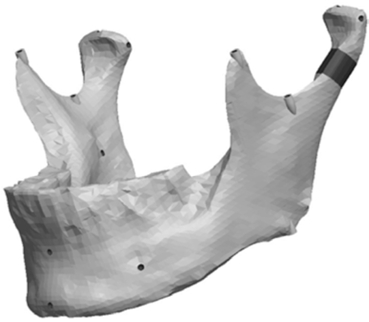
Model with a vertical condyle asymmetry.

**Fig. (11):**
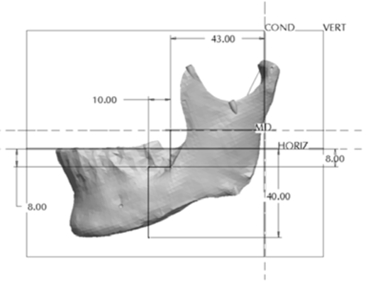
Location and design of the body asymmetry.

**Fig. (12):**
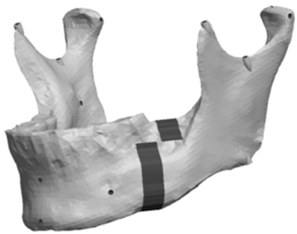
Model with a 9mm anteroposterior body asymmetry.

**Fig. (13):**
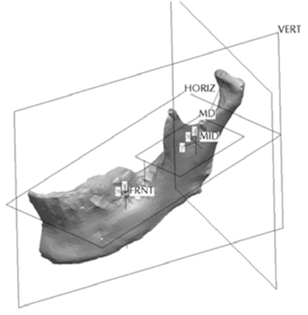
Location of the ramus asymmetries.

**Fig. (14):**
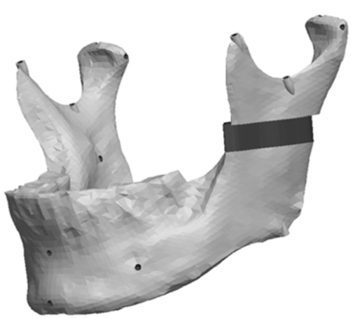
Model with a vertical ramus asymmetry.

**Fig. (15):**
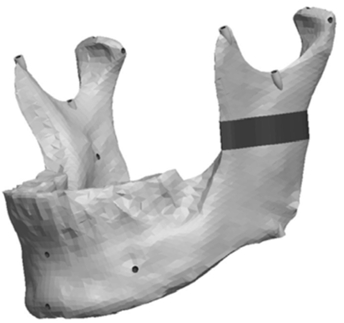
Model with a complex vertical and horizontal lateral ramus asymmetry.

**Table 1:** Anatomic Landmark Descriptions

Mandibular Landmarks	Description
**Ag**	Antigonial Notch
**B**	B-point
**Cl**	Lateral pole of Condyle head
**Cm**	Medial pole of Condyle head
**Co**	Superior point of Coronoid process
**Cp**	Chin Point
**Cs**	Superior position on Condyle head
**Dm**	Distal-gingival border of lower last molar
**Go**	Anthropometric Gonion
**Li**	Lingula
**Mf**	Mental Foramina
**Sn**	Sigmoid notch
